# Gastrointestinal and Liver Complications in Patients with Diabetes Mellitus—A Review of the Literature

**DOI:** 10.3390/jcm11175223

**Published:** 2022-09-04

**Authors:** Ruxandra Mare, Ioan Sporea

**Affiliations:** 1 Department of Internal Medicine II, Gastroenterology and Hepatology Unit, Advanced Research Center in Gastroenterology and Hepatology “Victor Babes” University of Medicine and Pharmacy, 30041 Timisoara, Romania; 2 Regional Center of Research in Advanced Hepatology, Academy of Medical Science, 30041 Timisoara, Romania

**Keywords:** diabetes mellitus, digestive complications, liver disease

## Abstract

The number of diabetes mellitus patients has increased over the last few years in developing countries, along with obesity and sedentary lifestyle. Besides macroangiopathy and microangiopathy, damage to the nerve fibers of the peripheral nervous system is the most common chronic complication of diabetes. Digestive complications in diabetic patients represent a consequence of diabetic autonomic neuropathy involving the gastrointestinal tract, but unfortunately not always evaluated by diabetologists. Aside from the complications encountered in the digestive tract, patients with diabetes mellitus are prone to developing liver diseases. This review will describe the prevalence of these complications, the modality of diagnosis, and therapeutical solutions in order to reduce the risk of progression of these complications in diabetic subjects.

## 1. Introduction

Diabetes is one of the diseases of the modern world, with an increasing prevalence in recent decades, especially in developed countries. The global prevalence of type 2 diabetes is estimated at 10.5% [1] which has increased during the last two decades in tandem with obesity and sedentary lifestyle.

The diabetologist carefully monitors the diabetic patient for the typical complications of this disease, such as micro and macroangiopathy. However, recently, mounting data have suggested that diabetic patients, particularly those with type 2 diabetes, may experience a variety of digestive complications, including those connected to gastrointestinal or hepatic disease. Gastrointestinal (GI) complications of diabetes are often caused by abnormal GI motility, which is a consequence of diabetic autonomic neuropathy involving the GI tract. Up to 75% of diabetes patients may experience GI symptoms, and this is a consequence of poor blood glucose control and not necessarily due to the duration of diabetes [2]. GI conditions caused by diabetes include gastroparesis and intestinal enteropathy.

Gastroparesis is a well-recognized GI manifestation of diabetes, most frequently encountered in women [3]. The prevalence of gastroparesis varies widely in specialized centers; up to 40% of patients with type 1 diabetes mellitus (T1DM) [4] and up to 30% of patients with type 2 diabetes mellitus (T2DM) [5] have gastroparesis. Enteropathy is a less recognized GI manifestation of diabetes and clinical presentation includes diarrhea, constipation, and fecal incontinence, which mainly is nocturnal [2]. Over the last few years, it was demonstrated that there is a bidirectional relationship between diabetes, mainly T2DM, and non-alcoholic fatty liver disease (NAFLD). The presence of NAFLD increases the incidence of T2DM and accelerates the development of complications in them, while T2DM increases the probability of the progression of non-alcoholic fatty liver to non-alcoholic steatohepatitis (NASH), cirrhosis, and hepatocellular carcinoma [6]. If an increase in the frequency of gallstones in diabetics has long been known and reported, other associations between diabetes mellitus and the presence of Helicobacter pylori and between diabetes and the risk of colon cancer are less known. Additionally, there is more and more evidence that there is a link between diabetes and hemochromatosis, and according to the latest guideline of the American Diabetes Association, all adult patients with T1DM should be screened for celiac disease in the presence of gastrointestinal symptoms, signs, or laboratory manifestations suggestive of celiac disease [7].

In this setting, this review focuses on the prevalence, clinical manifestation, evaluation, and management of this complication in order to provide a practical approach to this often under-recognized and challenging complication of diabetes.

## 2. Diabetic Autonomic Neuropathy of the Gastrointestinal Tract

Neuropathy of the gastrointestinal tract leads to the development of numerous complications, such as the exacerbation of gastroesophageal reflux disease (GERD), gastroparesis, and enteropathy.

### 2.1. Gastroesophageal Reflux Disease (GERD)

In diabetes patients, GERD is caused by abnormal peristalsis, spontaneous contractions, impaired lower esophageal sphincter (LES) tone, and an increased number of transient LES relaxation [8]. The clinical manifestations of GERD in this category of patients are pyrosis and regurgitation. The diagnosis of GERD can be based on clinical symptoms, and upper endoscopy or Ph-metry should be reserved only for cases that are resistant to treatment and in the presence of extra-esophageal symptoms. Treatment should focus on the normalization of body weight, the avoidance of coffee, alcohol, fried and roasted products, and proton pump inhibitors [9].

### 2.2. Diabetic Gastroparesis

Gastroparesis is characterized by delayed gastric emptying in the absence of mechanical obstruction [10]. Diabetic gastroparesis is thought to be caused by impaired vagal control, abnormal myenteric neurotransmission, the impairment of inhibitory nitric oxide-containing nerves, damage to the interstitial cells of Cajal, and underlying smooth muscle dysfunction [3,11]

The prevalence of diabetic gastroparesis varies between studies and between centers and this is due to the fact that most population-based studies have focused on symptoms rather than gastric scintigraphy findings. In such investigations, 5–12% of patients with diabetes report symptoms consistent with gastroparesis [2,12], and up to 20–40% of patients [9,13] have gastroparesis assessed by gastric emptying studies mostly performed in tertiary centers. The prevalence of gastroparesis is higher in women than in men, especially during the luteal phase of the menstrual cycle [14,15] mainly because gastric muscle contractility is reduced by progesterone. Patients with gastroparesis can present with early satiety, nausea, vomiting, bloating, postprandial fullness, upper abdominal pain, and, in severe cases, with weight loss [16]. The predominant symptom may vary based on the underlying etiology. For example, in a study that included 416 patients with gastroparesis, those with diabetic gastroparesis had more severe retching and vomiting as compared with patients with idiopathic gastroparesis [17]. Either way, gastroparesis should be suspected in diabetic patients presenting with this symptomatology and evaluation should begin with a history and physical examination, completed by laboratory studies (complete blood count, thyroid-stimulating hormone test, metabolic panel, amylase test if the patient has abdominal pain, and pregnancy test if appropriate) [11]. Furthermore, in order to rule out a mechanical obstruction, patients should undergo an upper gastrointestinal endoscopy, a computed tomographic enterography (CT), or a magnetic resonance (MR) enterography to exclude mechanical obstruction from a small bowel mass or superior mesenteric artery syndrome. In patients with suspected gastroparesis and no evidence of a mechanical obstruction on imaging or upper endoscopy, an assessment of gastric motility is mandatory to establish the diagnosis of gastroparesis. There are many tests that can be used for the assessment of gastric motility (Table 1), but gastric emptying scintigraphy is considered to be the gold standard and is recommended by the American Gastroenterological Association to confirm the diagnosis of gastroparesis [11]. The first-line treatment for diabetic gastroparesis should include dietary modifications, glycemic control, and the restoration of fluids and electrolytes but this nutritional approach will not be enough to control the symptoms of gastroparesis as the disease progresses. In addition to this, many patients will also require pharmacological, endoscopic, or surgical treatments. Table 2 summarizes the pharmacological, endoscopic, and surgical therapies available to treat gastroparesis.

### 2.3. Diabetic Enteropathy

Neuropathy of the intestinal tract leads to the development of numerous complications, such as diarrhea, habitual constipation, and fecal incontinence. Among these, the prevalence of diarrhea in diabetics is about 20% [25]. The mechanisms of diarrhea are very different. In the case of neuropathy, disturbances in intestinal peristalsis or disturbances in the transport of water and electrolytes are observed, which leads to watery diarrhea. Additionally, small intestinal bacterial overgrowth (SIBO) may result from abnormal small bowel motility. Diarrhea in patients with diabetic enteropathy is nocturnal, watery, and painless. Diarrhea can be associated with fecal incontinence caused by internal and external anal sphincter dysfunction, anorectal reflexes, and rectal motor-sensory dysfunction, and the diagnosis is based on the use of anorectal manometry [26]. Diarrhea in diabetics can be episodic, alternating with periods of normal intestinal transit or periods of constipation.

The treatment of diarrhea will depend on its etiology. Neuropathic forms may require the use of Loperamide, the use of opioid-based agents, and, in the event of severe refractory diarrhea, somatostatin analogues may be useful [27]. Bacterial overgrowth is found in approximately 44% of diabetic patients with diarrhea [28]; consequently, treatment should include the intermittent and maybe long-term administration of selective antibiotics (e.g., Rifaximin).

Constipation is another common complication of diabetes, affecting up to 60% of people [29]. The cause for constipation in this category of patients is due to a generalized slowing in the movement of the bowel [30]. Finally, the management of constipation consists of using traditional laxatives.

## 3. Non-Alcoholic Fatty Liver Disease and Diabetes

**Figure 1:**
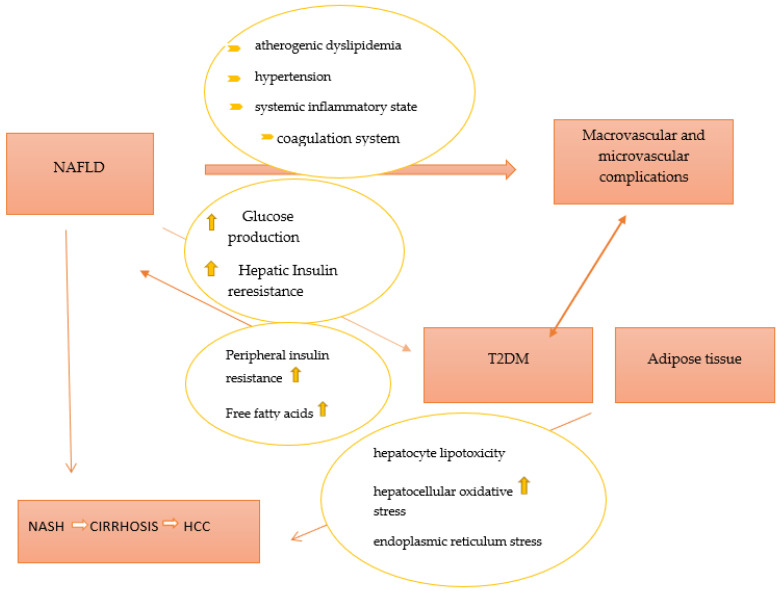
The pathophysiological link between NAFLD and T2DM. Abbreviations: T2DM—type 2 diabetes mellitus; NAFLD—non-alcoholic fatty liver disease; NASH—non-alcoholic steatohepatitis; HCC—hepatocellular carcinoma.

Inactivity and an imbalanced diet (high in fat and sugar content) are two major contributors to non-alcoholic fatty liver disease (NAFLD), one of the most prevalent causes of chronic liver disease. NAFLD is commonly classified into two phenotypes, nonalcoholic fatty liver (NAFL) and non-alcoholic steatohepatitis (NASH). Of these, NASH is associated with an increased risk of hepatic morbidity and mortality due to the risk of the development of severe fibrosis, cirrhosis, and hepatocellular carcinoma [31]. Previously, NAFLD has been considered as a hepatic component of metabolic syndrome (MetS), but recently, an association between NAFLD and type 2 diabetes mellitus has been described [32]. Insulin resistance, especially in adipose tissue and liver, lipotoxicity, and inflammation, is part of the common pathophysiological mechanisms of NAFLD and T2DM. Over the years, it has been demonstrated that NAFLD contributes to the development of T2DM by increasing hepatic glucose production and exacerbating hepatic insulin resistance as a result of the activation of hepatic protein kinases Cε and liver-secreted proteins with diabetogenic properties, such as fetuin A, fetuin B, RBP4, selenoprotein P, DPP4, and HFREP1 [33,34,35,36,37,38]. Furthermore, intrahepatic fat accumulation activates liver inflammation and further promotes the development of atherogenic dyslipidemia (an increase in small and dense lipoprotein particles -LDL, triglycerides, and a decrease in HDL cholesterol) and hypertension (activation of the renin–angiotensin–aldosterone system). Additionally, it triggers a systemic inflammatory state (increased protein C reactive, Interleukin 6, tumor necrosis factor, and reactive oxygen species) as well as a coagulation mechanism (increased fibrinogen, factor VII, and PAI-1) [39]. All procedures play important roles in the development of diabetic macrovascular and microvascular complications. On the other hand, T2DM and systemic insulin resistance promote an increase in the flux of free fatty acids from peripheral tissues to the liver, leading to the development and progression of NAFLD. Moreover, T2DM promotes the development of NAFLD through a number of mechanisms, such as direct hepatocyte lipotoxicity, hepatocellular oxidative stress brought on by an increase in the oxidation of free fatty acids, endoplasmic reticulum stress, causing the release of inflammatory cytokines by hepatic Kupffer cells and peripheral adipocytes, and hepatocellular apoptosis and necrosis, respectively [39]. A better representation of the relationship between T2DM and NAFLD is presented in Figure 1.

Two recent reviews explain the bidirectional relationship between NAFLD and insulin resistance and underline the clinical implications associated with these two conditions on the cardiovascular (CV), renal, and peripheral nervous systems. [40,41]. A meta-analysis revealed that patients with NAFLD had a greater risk of fatal and/or non-fatal CV events by up to 1.64 times (95% CI 1.26–2.13) than those without NAFLD [42]. Additionally, patients with T2DM and NAFLD have been found to have a higher prevalence of coronary, cerebrovascular, and peripheral vascular disease than those without NAFLD [43]. Similarly, to the association between NAFLD and coronary heart disease, several studies have studied the association between chronic kidney disease (CKD) and NAFLD [44,45]. A recent meta-analysis found a risk of CKD in NAFLD patients 1.43 times higher, even after adjustment for other factors such as age, sex, obesity, hypertension, and diabetes [44]. Another study [45] that included 4746 patients showed that liver fibrosis was associated with an increased prevalence of albuminuria and CKD.

While the association of T2DM with its microvascular and macrovascular complications is well established, the association of T2DM with NAFLD is more recently recognized. Furthermore, because patients are usually asymptomatic and routine blood tests are often normal, it may be an overlooked diagnosis in patients with T2DM. T2DM is one of the strongest clinical predictors of NAFLD progression to NASH and liver cirrhosis. The presence of diabetes increases the risk of NASH two to three times [46]. A recent study performed on T2DM patients using liver biopsy revealed that NASH was present in 96.8% of patients with T2DM, suggesting that the latter might be one of the early complications encountered in T2DM patients due to its pathophysiological correlation with insulin resistance [47].

The prevalence of diabetes in patients with NAFLD and NASH is estimated to be 22.5% and 43.6% [48], respectively, which is much higher than the prevalence of diabetes in the general population (8.5%), while the prevalence of NAFLD and NASH among patients with T2DM is 55.5% and 37.3% [49]. Nowadays, few data are available regarding the prevalence of NAFLD in people with T1DM. Some studies reported that ultrasound-diagnosed NAFLD was present in nearly 20–30% of adult patients with T1DM [50,51]. Clinically, patients will not have any symptoms besides fatigue, but in advanced stages due to the development of liver cirrhosis, they can develop ascites, esophageal varices, and jaundice. NAFLD is defined as the presence of hepatic steatosis, documented either by imaging or by histology, in the absence of significant alcohol consumption, the long-term use of steatogenic medication, or hereditary disorders [52]. Over the years, in the absence of histology, the presence of NASH was considered when patients had increased levels of transaminases, but recent studies have shown that approximately 56% of individuals with histologically proven NASH have normal liver enzymes [53,54].

Furthermore, liver fibrosis appears to be the main factor influencing the long-term survival of these patients. A meta-analysis published in the literature reported that the prevalence of advanced liver fibrosis quantified by liver biopsy in patients with type 2 DM is up to 17% [49]. A more recent meta-analysis [55] showed that the prevalence of elevated liver stiffness diagnosed by Vibration Controlled Transient Elastography (VCTE) in adult patients with T1D was 5.2% and 19.8% in patients with T2D. Based on this premise, guidelines such as the American Diabetes Association (ADA) [7], the European Association for the Study of Diabetes (EASD), the European Association for the Study of the Liver (EASL), and European Association for the Study of Obesity (EASO) [56], recommend the screening of fibrosis in diabetic patients using biomarkers and/or VCTE, with some studies suggesting the use of VCTE in this population, given the lower accuracy of biochemical tests [57]. Over the years, many noninvasive scores that could predict the presence of advanced fibrosis using routinely available labs and demographic data have been developed. Table 3 provides a summary of the most widely used fibrosis scores in patients with NAFLD.

**Figure 2:**
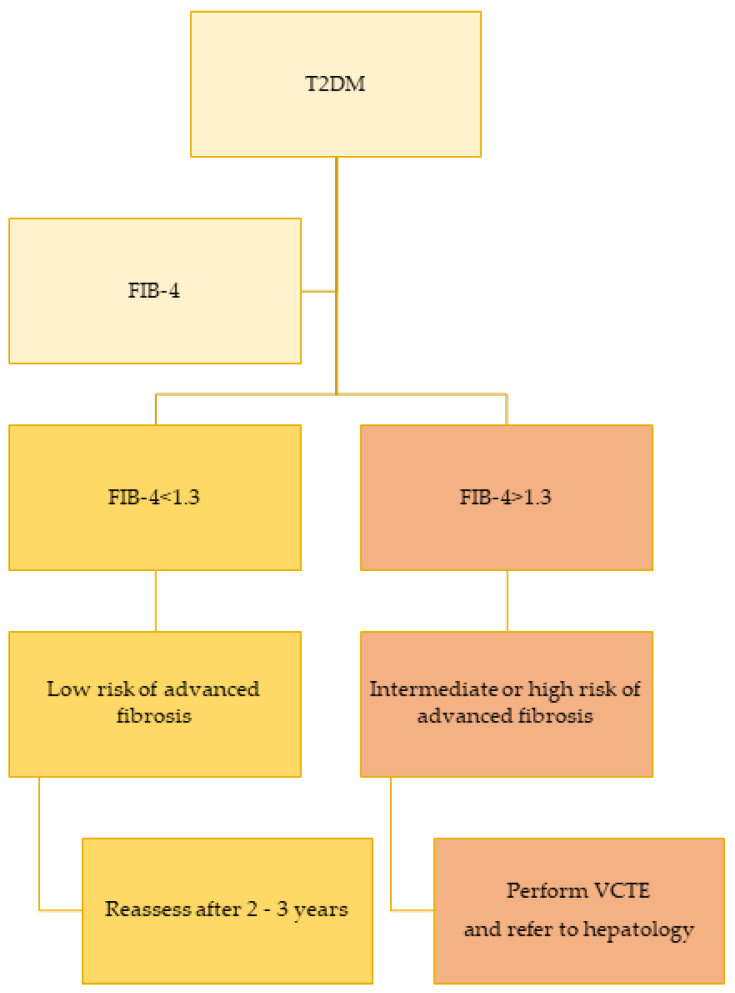
Proposed algorithm to screen patients with type 2 diabetes. Abbreviations: T2DM—type 2 diabetes mellitus; FIB-4—fibrosis index-4; VCTE—vibration controlled transient elastography.

These scores (FIB-4 is among the best studied) have reasonable specificity and can be practical for healthcare providers to assess patients with suspected NAFLD based on ultrasound or elevated levels of ALT. A recent study analyzed the impact of a “FIB-4 first” strategy to reduce the need for VCTE and hepatology referral [62]. The use of FIB-4 and VCTE in a staged risk-stratification approach could prevent up to 87% of additional examinations [62]. Figure 2 provides an algorithm for liver fibrosis screening in patients with T2DM.

Currently, NAFLD-specific pharmacologic therapies approved for widespread use are limited, and the ones that do show beneficial effects are available in randomized clinical trials. Diet and lifestyle changes remain the main treatments for NAFLD. It seems that more than 7% of weight loss is associated with histological improvement [56]. In addition to this, Pioglitazone is currently recommended by EASL in selected individuals [56] with NAFLD and T2D. The glucagon-like peptide-1 (GLP-1) receptor agonist (liraglutide) has also been reported to determine the resolution of NASH without fibrosis worsening [63]. Sodium-glucose cotransporter 2 (SGLT-2) inhibitors [64] reported improvement in liver enzymes, liver steatosis, and even liver histology in randomized clinical trials. Moreover, NAFLD is associated with an increased risk of developing hepatocellular carcinoma (HCC), particularly among those who have cirrhosis or advanced fibrosis, but 20–30% of NAFLD-associated HCC cases occur in the absence of advanced fibrosis [65]. The link between T2DM and HCC is mediated by a chronic inflammatory state. Recent research has shown that eradicating HCV infection with direct-acting antivirals (DAAs) reduces the chronic inflammatory state, which in turn delays the formation of type 2 diabetes [66].

Redefining non-alcoholic fatty liver disease (NAFLD) as metabolic dysfunction-associated fatty liver disease (MAFLD) was recently suggested by international experts [67]. The experts came to the conclusion that the term NAFLD does not accurately reflect current knowledge, and the metabolic term “MAFLD” would be much more accurate and correct. The diagnosis of MAFLD requires the presence of fatty liver in addition to any of the following: overweightness/obesity, type 2 diabetes mellitus (T2DM), or evidence of metabolic dysregulation. In a study that included 7,761 individuals, MAFLD was associated with an increased risk of all-cause mortality, while NAFLD demonstrated no relationship with all-cause mortality after adjusting for metabolic risk factors [68]. As a result, the authors concluded that alcoholic fatty liver disease (NAFLD) is a component of a larger multi-system disease that also includes obesity, diabetes, high blood pressure, and high cholesterol. Additionally, they suggested that changing the name of NAFLD to MAFLD may help clinicians better understand the factors that raise the risk of death. In a more recent study, the authors created a simplified set of metabolic syndrome (MetS)-based criteria for MAFLD and compared the performance of the reduced criteria with that of the original criteria in predicting all-cause mortality in a 27-year follow-up of American adults [69]. The authors combined BMI and waist circumference as one indicator of obesity/central obesity in the simplified criteria; they also combined T2DM and prediabetes indicators into one criterion rather than listing them as separate categories and did not use HOMA-IR score and high-sensitivity C-reactive protein either, but they included hyperuricemia in the simplified criteria. Finally, the conclusion of the study was that the simplified MAFLD criteria may better identify high-risk individuals in clinical practice [69], but further studies are needed until this nomenclature is accepted by all international societies.

## 4. Association between Diabetes and Other GI Diseases

### 4.1. Diabetes and Gallbladder Stones

Nowadays, there is increasing evidence that metabolic syndrome [70], insulin resistance [71], and overweightness and obesity [72] are associated with increased gallbladder disease. Gallstones are more common in diabetics than in non-diabetics. Thus, an Italian study [73] evaluating a cohort of 1337 diabetics, of which 1235 were T2DM and 102 were T1DM, respectively, found a significantly higher prevalence in diabetics than in non-diabetics (24.8% compared to 13.8%, *p* = 0.0001). In this study, the prevalence of gallstones was higher in women compared to men (29% vs. 22%, *p* = 0.003) and increased with age (13% under the age of 40 and 30%, in patients > 65 years) and body mass index (24% in non-obese and 30% in obese patients (*p* = 0.001)). At the same time, this study did not find a relationship between the type of diabetes and the frequency of gallstones.

Similar results were obtained in two published meta-analyses [74,75], where the risk of developing gallstones in diabetics compared to the non-diabetic population was increased up to 1.75 times. In another meta-analysis [76], the risk of gallbladder cancer in diabetics was increased by approximately 1.5 times compared to non-diabetics. Furthermore, another meta-analysis [77] demonstrated that diabetic patients have a 1.74-fold increased risk of developing acute pancreatitis compared to non-diabetics. Additionally, one publication found that the severity and risk of complications and death in acute pancreatitis are higher in diabetics compared to non-diabetics [77].

In this clinical setting, should we perform on all diabetic patients an ultrasound to discover the presence of gallstones in order to make the best medical decision?

Authors should discuss the results and how they can be interpreted from the perspective of previous studies and of the working hypotheses. The findings and their implications should be discussed in the broadest context possible. Future research directions may also be highlighted.

### 4.2. Diabetes and Helicobacter pylori Infection

The relationship between diabetes and chronic Helicobacter pylori (HP) infection has been discussed for a long time, but some more recent meta-analyses have attempted to prove this relationship. Studies have shown that HP can promote insulin resistance by inducing chronic inflammation and affecting the insulin regulation of gastrointestinal hormones [78]. Thus, a meta-analysis published in 2013 [79], which included 14,080 diabetic patients, found a prevalence of HP infection of 42.2%. The risk of HP infection was increased in diabetics vs. non-diabetics by 1.33 times (*p* = 0.008), while in T2DM it was increased by 1.76 times (*p* < 0.00001). Another meta-analysis published in 2020 [80] that analyzed 41 studies with 9559 individuals found an increased frequency of HP infection in diabetics compared to non-diabetics by 1.27 times (1.19 times in T1DM and 1.43 times in T2DM).

Starting from these premises, the question arises whether it would not be necessary to eradicate HP infection in diabetics, which could perhaps improve carbohydrate metabolism and insulin resistance and allow a more favorable evolution of the disease (perhaps only in DM type 2)?

### 4.3. Diabetes and Colorectal Cancer

Diabetes is associated with a higher risk of colorectal cancer (CRC). In a meta-analysis [81], CRC patients with diabetes had a significantly increased risk of all-cause mortality up to 1.17 times, cancer-specific mortality up to 1.12 times, and had worse disease-free survival up to 1.54 times, compared to CRC patients without diabetes. In a study published in 2020 on a large cohort [82], patients diagnosed with diabetes mellitus before the age of 50 had a 1.9 times increased risk of developing CRC, while a diagnosis of diabetes after the age of 50 years increased the risk up to 1.3 times. On the other hand, a diagnosis of diabetes before the age of 50 in those with a family history of CRC was associated with a 6.9-fold increased risk of colon cancer, and only a 1.9-fold increase in those diagnosed after the age of 50.

Furthermore, a recent study [83] demonstrated an increased risk of interval cancers in diabetics compared to the general population. Thus, in this retrospective Danish study performed on a large cohort of diabetic and non-diabetic patients who underwent colonoscopy, the rate of interval CRC was 0.64% in diabetics and 0.36% in non-diabetics.

These recent studies could change the colon cancer screening strategy in diabetic patients, either with an earlier start of screening in this category or by shortening colonoscopy screening intervals. The mechanism by which the risk of CRC seems to be increased in diabetics may be related to the coexistence of associated metabolic factors (obesity, hypercholesterolemia) or possible changes in the microbiota in these patients.

### 4.4. Diabetes and Hemochromatosis

The prevalence of idiopathic hemochromatosis is 9.6 per 1000 in persons with diabetes versus 4 per 1000 persons in the general population [84]. Iron overload in the body causes severe damage to the beta-cells through excessive oxidative stress. In addition, the ability to use insulin and gluconeogenesis in the liver is weakened, resulting in the occurrence and development of type 2 diabetes [85]. A recent meta-analysis [86] showed that the risk of type 2 diabetes increased with the increase in serum ferritin concentration up to 1.20 times. Consistent with the current results, three meta-analyses [87,88,89] showed that high ferritin levels were associated with the risk of type 2 diabetes.

### 4.5. Diabetes and Celiac Disease

Over the years, it has been shown that there is an association between celiac disease (CD) and T1DM due to the fact that both T1DM and CD are autoimmune diseases that share a common genetic background (HLA DQ2 and 8 susceptibility). The mean prevalence of CD in patients with T1D is about 8% [90] while the prevalence of CD in T2DM is the same [91] or even lower than in the general population [92]. One study [93] showed that the prevalence of CD among patients with T2DM with poor glycemic control despite insulin therapy is slightly higher than the actual CD prevalence in the general population, underlying the necessity in this category of patients for further investigations. Due to the significantly higher prevalence of CD in T1DM, the American Diabetes Association recommends screening for CD in this category of patients [7] in the presence of gastrointestinal symptoms (diarrhea, bloating, and weight loss) and abnormalities(growth failure in children, osteoporosis, vitamin deficiencies, and iron deficiency anemia). In the majority of cases, T1DM is diagnosed before CD but it can happen the other way around. Screening for CD relies on highly sensitive and specific serology tests such as tissue transglutaminase (tTG) IgA, endomysial (EMA) IgA, and deaminated gliadin peptide (DGP) IgA and IgG antibodies, especially if IgA deficient [90]. The highest diagnostic yield is given by performing a duodenal biopsy [94]. Finally, a strict gluten-free diet is recommended in those with serological and histological evidence of CD.

## 5. Conclusions

Given the multitude of digestive complications among diabetic patients, the attention of the gastroenterologist has increased in this field, underlining the need for systematical screening for various pathologies in this category of subjects. A tight collaboration between gastroenterologists and diabetologists is indispensable for visible therapeutic results and for the development of common medical practice guidelines.

## Figures and Tables

**Table 1:** Tests used for the assessment of gastric motility.

Tests	Comments
Scintigraphic gastric emptying	The gold standard, most cost-effective, simple, and available technique able to assess liquid and solid emptying; minimal radiation exposure [9]
Wireless motility capsule	Measures simultaneously phasic pressure amplitudes, temperature, and Ph as it passes through the GI tract [18]
13 C breath testing	Non-invasive, non-radiation exposure. Acetate breath testing, octanoic acid breath test, or spirulin have been used to assess gastric emptying [19].
Electrogastrography	Noninvasive method that measures gastric myoelectrical activity [20].
Antroduodenal manometry	Invasive procedure requiring expertise to perform and interpret. Assess fasting and postprandial phases [21].

**Table 2:** Treatment options for gastroparesis.

Treatment	Mechanism	Comments
Metoclopramide (10 mg four times daily)	Improves gastric emptying by enhancing gastric antral contractions and decreasing postprandial fundus relaxation	First line therapy Symptoms improved in 25 to 62% of patients [11] Risk of tardive dyskinesia
Domperidone (10 mg three times daily)	Similar with Metoclopramide	Used when symptoms fail to respond to Metoclopramide Risk of cardiac arrhythmias [22]
Erythromycin (250 mg three times daily)	Motilin receptor agonist Induces high amplitude gastric propulsive contractions that increase gastric emptying	Used when symptoms fail to respond to Metoclopramide and Domperidone Duration: no more than 4 weeks Risk of tachyphylaxis [23]
Tricyclic agents	Reduce perception of pain at different levels of the brain–gut axis	Medication for visceral pain [23]
Gastric per-oral endoscopic myotomy (G-POEM)	Induces dumping syndrome	Pooled analysis including open-label and retrospective studies suggest a reduction in post-procedure GCSI scores and improved gastric emptying, with 6.8% overall adverse events Indication: only in refractory gastroparesis in tertiary centers [23]
Gastric electrical stimulation	Electric stimulation with high-energy, long-duration pulses	Reserved for compassionate treatment in patients with refractory symptoms (e.g., nausea and vomiting, without pain) [23]
Surgery	Pyloroplasty, gastrectomy	Most studies are non-randomized, unblended, or case series [11,24]

**Table 3:** Biomarkers for fibrosis staging.

Biomarker	Formula	Cut-Offs to Rule Out/in Advanced Fibrosis
FIB-4 index [58]	Age (years) × AST (U/L)/ [PLT (10^9^/ L) × ALT^1/2^ (U/L)	<1.3/>2.67
NAFLD fibrosis score [59]	−1.675 + 0.037 × age (years) + 0.094 × BMI (kg/m^2^) + 1.13 × IFG/diabetes (yes = 1, no = 0) + 0.99 × AST/ALT ratio − 0.013 × platelet count (× 10^9^/L) − 0.66 × albumin (g/dL).	<−1.455/>0.676
Enhanced liver fibrosis test (ELF) [60]	Age, hyaluronic acid, aminoterminal propeptide of type III collagen, and tissue inhibitor of matrix metalloproteinase 1	≥9.8
Alanine aspartate transferase (AST) to platelet ratio index (APRI) [61]	[(AST/ upper limit of the normal AST range)/platelet count (10^9^/L)] × 100	<0.5/>1.5

Abbreviations: BMI—Body mass index, IFG—Impaired fasting glucose, AST—Aspartate aminotransferase, ALT—Alanine aminotransferase, FIB—Fibrosis index, PLT—platelet count.

## Data Availability

Not applicable.
